# Pressure-Regulated Gene Expression and Enzymatic Activity of the Two Periplasmic Nitrate Reductases in the Deep-Sea Bacterium *Shewanella piezotolerans* WP3

**DOI:** 10.3389/fmicb.2018.03173

**Published:** 2018-12-21

**Authors:** Xue-Gong Li, Wei-Jia Zhang, Xiang Xiao, Hua-Hua Jian, Ting Jiang, Hong-Zhi Tang, Xiao-Qing Qi, Long-Fei Wu

**Affiliations:** ^1^Laboratory of Deep Sea Microbial Cell Biology, Institute of Deep-Sea Science and Engineering, Chinese Academy of Sciences, Sanya, China; ^2^International Associated Laboratory of Evolution and Development of Magnetotactic Multicellular Organisms, CNRS-Marseille/CAS, Sanya, China; ^3^State Key Laboratory of Microbial Metabolism, School of Life Sciences and Biotechnology, Shanghai Jiao Tong University, Shanghai, China; ^4^State Key Laboratory of Ocean Engineering, School of Naval Architecture, Ocean and Civil Engineering, Shanghai Jiao Tong University, Shanghai, China; ^5^University of Chinese Academy of Sciences, Beijing, China; ^6^Aix Marseille Université, CNRS, LCB, Marseille, France

**Keywords:** *Shewanella piezotolerans* WP3, nitrate reduction, periplasmic nitrate reductase (NAP), high hydrostatic pressure, piezotolerance

## Abstract

*Shewanella* species are widely distributed in marine environments, from the shallow coasts to the deepest sea bottom. Most *Shewanella* species possess two isoforms of periplasmic nitrate reductases (NAP-α and NAP-β) and are able to generate energy through nitrate reduction. However, the contributions of the two NAP systems to bacterial deep-sea adaptation remain unclear. In this study, we found that the deep-sea denitrifier *Shewanella piezotolerans* WP3 was capable of performing nitrate respiration under high hydrostatic pressure (HHP) conditions. In the wild-type strain, NAP-β played a dominant role and was induced by both the substrate and an elevated pressure, whereas NAP-α was constitutively expressed at a relatively lower level. Genetic studies showed that each NAP system alone was sufficient to fully sustain nitrate-dependent growth and that both NAP systems exhibited substrate and pressure inducible expression patterns when the other set was absent. Biochemical assays further demonstrated that NAP-α had a higher tolerance to elevated pressure. Collectively, we report for the first time the distinct properties and contributions of the two NAP systems to nitrate reduction under different pressure conditions. The results will shed light on the mechanisms of bacterial HHP adaptation and nitrogen cycling in the deep-sea environment.

## Introduction

Nitrogen is one of the building blocks of life and occurs naturally throughout the planet (Brandes et al., 2007; Denk et al., 2017; Kuypers et al., 2018). It forms numerous compounds with different chemical valences, and chemical transformations among them constitute the network of global nitrogen biogeochemical cycles (Stein and Klotz, 2016). Being one of the most stable nitrogen compounds, nitrate can be retained in soils, sediments, and seawater (Sparacino-Watkins et al., 2014). In the ocean, the concentration of nitrate increases from 0.22 μM at the surface to approximately 40 μM at the Challenger Deep, suggesting its important role in nitrogen cycling in the deep biosphere (Nunoura et al., 2015).

Due to its relatively high redox potential (*E*^0′^ = +433 mV), nitrate is believed to be the preferable terminal electron acceptor for microbial anaerobic respiration (Lam and Kuypers, 2011). Bacterial nitrate reductases catalyzing this reaction are classified into three types based on cellular location and physiological function (Stolz and Basu, 2002). The assimilatory nitrate reductase (Nas) is a soluble cytoplasmic protein that incorporates nitrogen from nitrate into the organism’s biomass (Kilic et al., 2017), while the membrane-bound (Nar) and the periplasmic (Nap) dissimilatory nitrate reductases (Nar and Nap) excrete the end products of nitrate reduction out of the cells.

A typical NAP system is made up of at least four subunits: NapA, B, C, and D (Gao et al., 2009). The catalytic subunit, NapA, contains two redox active centers: a molybdenum active site and an iron sulfur cluster. The reduction of nitrate takes place at the molybdenum center (Mintmier et al., 2018). NapB is a small c-type cytochrome that contains two heme molecules, and it delivers electrons to NapA (Kern et al., 2007; Jin et al., 2016). NapC is a transmembrane tetra-heme protein belonging to the NapC/NirT family, which is believed to transport electrons via heme cofactors from the membrane quinone pool to the periplasmic NapB (Wei et al., 2016). The chaperone protein NapD is generally considered to be involved in NapA maturation by binding to its N-terminal TAT leader sequence (Sparacino-Watkins et al., 2014). Apart from the core components mentioned above, some bacteria also have auxiliary proteins, such as NapEGH (Brondijk et al., 2004; Kern and Simon, 2009).

The regulation of *nap* operons is under the control of many factors. NarQ-P is a typical two-component system for *nap* regulation. NarQ senses the presence of nitrate and phosphorylates the response regulator NarP, which activates the transcription of the *nap* operon (Stewart, 2003). The global transcriptional regulators EtrA and CRP are also required for the expression of *nap*. Studies in *Shewanella oneidensis* MR-1 showed that deletion of *etrA* and *crp* obviously reduces or completely suppresses the expression of *nap* (Cruz-Garcia et al., 2011; Dong et al., 2012). In addition, alteration of the cellular redox state by temperature, oxygen, or carbon sources also influences the expression of the *nap* operon (Wang and Chen, 2015).

*Shewanella* is a genus of facultative anaerobic bacteria widely distributed in marine and freshwater environments. They are capable of utilizing a wide variety of terminal electron acceptors and surviving in varying environments (Hau and Gralnick, 2007). Systematic genomic surveys identified two types of NAP systems in the genus of *Shewanella*. Strains inhabiting over 5,000 m depths generally harbor only the NAP-α operon (*napEDABC*), some shallow water strains possess only the NAP-β operon (*napDAGHB*), and those dwelling in the middle water layer encode the two NAP systems simultaneously (Simpson et al., 2010; Chen et al., 2011). It is thus proposed that the two NAP systems have different physiological functions, and NAP-α may be preferable in the deep-sea high-pressure environment. Yet the differences between the two NAP systems and their functions, especially under high hydrostatic pressure (HHP) conditions, remain unknown.

*Shewanella piezotolerans* WP3 was isolated from sediment of the west Pacific at the depth of 1,914 m, and its whole genome has been sequenced (Xiao et al., 2007; Wang et al., 2008). The ranges of temperature and pressure for the growth of WP3 are 0–35°C and 0.1–50 MPa, respectively. Previous studies show that WP3 possesses both *nap-α* (*napD1A1B1C*) and *nap-β* (*napD2A2B2*) operons, but not the Nas or Nar systems (Chen et al., 2011). Moreover, both NAP systems are functional during denitrification at 0.1 MPa (Chen et al., 2011). WP3 provides a favorable experimental model for the study of NAP systems under HHP. In this study, by measuring cell growth and nitrate utilization, we showed that single *napA* deletion mutants were able to grow by nitrate anaerobic respiration at a level comparable to the wild-type strain harboring both systems under HHP conditions. However, enzymatic and gene expression analysis suggested the two NAP systems differed in piezotolerance and were regulated through different but correlated regulation pathways. Our results suggested that possessing redundant respiration machinery with a distinct response to HHP might be an adaptive strategy for WP3 to cope with HHP in the deep-sea environment.

**Table 1:** Strains used in this study.

Strains	Description	Reference
***S. piezotolerans* WP3**		
WT	Wild type	Xiao et al., 2007
WP3-α	*napA2* deletion mutant derived from WP3	Chen et al., 2011
WP3-β	*napA1* deletion mutant derived from WP3	Chen et al., 2011
Δ*napA1*Δ*napA2*	*napA1* and *napA2* double deletion mutant derived from WP3	Chen et al., 2011

## Materials and Methods

### Bacterial Strains and Growth Conditions

The strains used in this study are listed in Table 1. *S. piezotolerans* WP3 was cultured microaerobically in 2216E broth (Difco, United States) at 20°C. To investigate the growth of WP3 and derived mutants under HHP, each culture was grown in 2216E media at atmospheric pressure to stationary phase and then diluted to an optical density of 0.01 at 600 nm (Cary 60, UV-Vis, Agilent Technologies). When necessary, 4 mM nitrate and 20 mM lactate were added to the media. For the HHP growth experiments, the cells were cultivated in 2.5 ml disposable syringes without air. Then, a combi stopper (B. Braun, Melsungen, Germany) was used to replace the needle to insulate the media. The prepared syringes were placed inside stainless-steel high-pressure vessels (Feiyu Science and Technology Exploitation Co., Ltd., Nantong, China), and the hydrostatic pressure was applied using a water pump (Top Industrie, France).

### RNA Extraction and cDNA Synthesis

Briefly, the 1.5 ml mid-exponential phase culture was centrifuged at 15,294 *g* and 4°C, and the cell precipitate was collected. Total RNA was isolated with a TRI reagent-RNA/DNA/protein isolation kit (Molecular Research Center, Inc.) according to the manufacturer’s instructions. The RNA samples were purified by digesting the residual DNA fragments with DNase I at 37°C for 1 h and quantified using the NanoDrop 2000 spectrophotometer (Thermo Fisher Scientific, Waltham, MA, United States). For cDNA synthesis, the PrimeScript^TM^ II 1st strand cDNA synthesis kit (TAKARA, Shiga, Japan) was used, and cDNA was synthesized under the reverse transcription of PrimeScript II RTase.

### Real-Time PCR (RT-PCR)

The quantification of *napA* transcript was performed by RT-PCR as described previously (Jian et al., 2012) with some modifications. Two pairs of primers, napA1-S/R and napA2-S/R, were used to quantify the relative transcript abundance of *napA1* and *napA2* (Chen et al., 2011). Real-time PCR was performed using an Applied Biosystems StepOnePlus^TM^ Real-Time PCR system and Power SYBR Green PCR Master Mix (Applied Biosystems). The RT-PCR was performed using StepOne Software (ABI) in reaction mixtures with total volumes of 20 μl containing 10 μl FastStart Universal SYBR Green Master Mix (Rox) (Roche, Mannheim, Germany), 0.5 μM of each primer and 1 μl cDNA template. The amount of target was normalized to that of the reference gene *swp2079*. Every experiment was performed in triplicate for each sample, and a mean value and standard deviation were calculated for the relative RNA expression levels. The transcript abundance was determined using the standard curve method. Each assay was performed in triplicate, and a mean value and standard deviation were calculated.

### Preparation of the Periplasmic Fraction

The periplasmic fraction was prepared according to the method described previously (Yin et al., 2018). The mid-exponential phase cells of WP3 were harvested by centrifugation at 15,294 *g* and 4°C. The cell pellet was re-suspended with Tris–HCl buffer (20 mM, pH 7.4), with Complete Protease Inhibitor added according to the preparation note (Roche, Mannheim, Germany). Then, a proper volume of chloroform was added, and the solution was gently mixed and incubated at room temperature for 5 min. Then, 10 times volume of Tris–HCl buffer was added to the tube, and the tube was rotated 2–3 times. After centrifugation at 15,294 *g* and 4°C for 5 min, the supernatant aqueous phase was transferred into a clean tube, and centrifugation was repeated. The protein content was measured with a Pierce BCA Protein Assay Kit (Thermo Fisher Scientific, Rockland, ME, United States).

### Nitrate Concentration Determination

Nitrate concentration during growth was monitored using the Szechrome NAS reaction (Polysciences, Inc.). Briefly, equal volumes of analytical grade concentrated phosphoric acid (85–86%) and concentrated sulfuric acid (95–97%) were mixed and stood in closed flasks for at least 1 week. Then, 1 vial (5 g) NAS was dissolved in a liter of a mixture of equal volumes of nitrate-free concentrated H_3_PO_4_ and concentrated H_2_SO_4_. For nitrate determination, a 10-fold diluted sample was gently mixed with reagent solution, and violet color intensity was read at 570 nm. A nitrate standard curve was generated to convert absorbance values to concentrations.

### Assays for Nitrate Reductase Activity

The nitrate reductase activity of the obtained periplasmic fraction was assayed using the modified protocol (Ikeda et al., 2009; Yin et al., 2018). The Tris–HCl buffer was bubbled with N_2_ at least 15 min, then the methyl-viologen, nitrate, and Na dithionite were added until a dark blue color appeared. The 2.5 ml disposable syringes were used to distribute the reaction mixture in 2 ml aliquots, and a stopper was used to avoid air leaks. In the case of the measurement of the activity under high pressure, the syringes were placed inside stainless-steel high-pressure vessels (Feiyu Science and Technology Exploitation Co., Ltd., Nantong, China), and the hydrostatic pressures were achieved using a water pump (Top Industrie, France). After incubation at room temperature for 20 min, syringes were shaken vigorously until the blue color disappeared. The produced nitrite was measured by the diazo-coupling method as described previously (Chen et al., 2011).

## Results

### NAP Systems Are Capable of Catalyzing Nitrate Reduction Under HHP Condition

**FIGURE 1:**
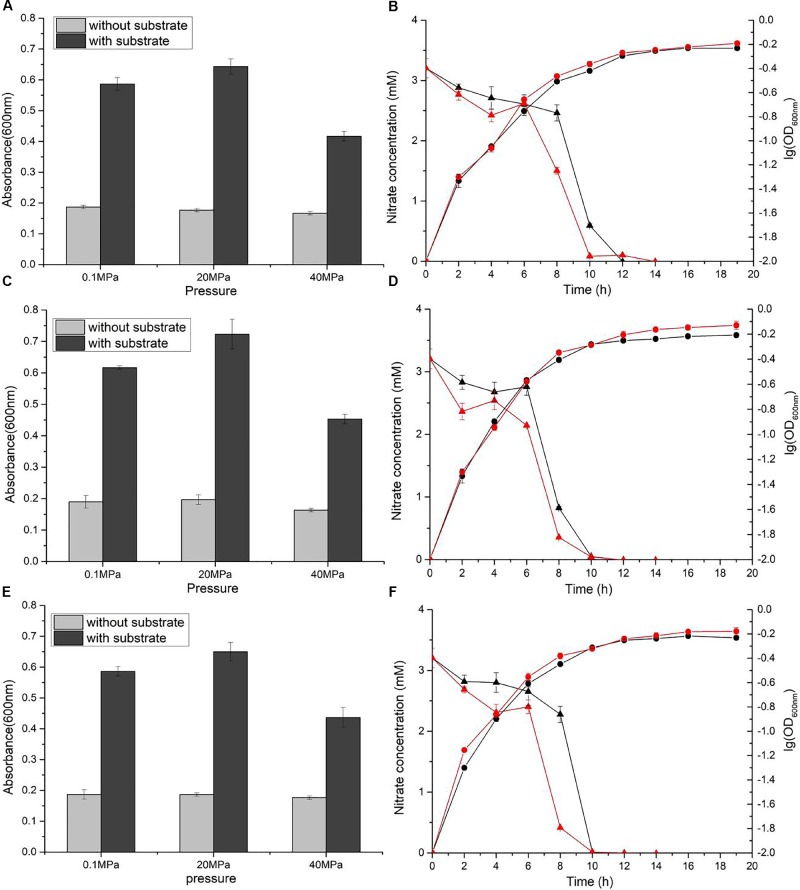
Nitrate sustained growth of WP3 and Δ*napA* mutants under different pressures. Final biomass of WP3 **(A)**, WP3-α **(C)** and WP3-β **(E)** cultivated in 2216E media with or without substrate (nitrate plus lactate) at different pressure conditions. Growth curve and nitrate consumption curve of WP3 **(B)**, WP3-α **(D)** and WP3-β **(F)** at 0.1 and 20 MPa. Each value was the average of three measurements. •: cell density at 0.1 MPa; 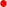: cell density at 20 MPa; 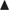: nitrate concentration of the supernatant at 0.1 MPa; and 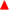: nitrate concentration of the supernatant at 20 MPa.

Nitrate reduction in WP3 has been extensively studied under atmospheric pressure conditions, but its characteristics under HHP conditions close to its original habitat remain unknown. To assess the nitrate respiration of WP3 at HHP, cells were cultivated in 2216E medium at 0.1, 20, and 40 MPa, with or without the supplementation of nitrate, and the cell density (represented by OD_600nm_) was measured. It has been reported that nitrate and lactate serve, respectively, as an electron receptor and donor and improve the WP3 growth (Chen et al., 2011). As expected, the growth of WP3 stayed at a relatively low level under all pressure conditions when substrate (nitrate and lactate) was absent. In contrast, the addition of substrate obviously promoted cell growth, though to different extent depending on the pressures applied (Figure 1A). At atmospheric pressure, the final biomass increased approximately 3.1 times (OD_600nm_: 0.59 ± 0.02 versus 0.19 ± 0.01). When cultivated at 20 MPa, close to the ambient pressure condition where WP3 was isolated, the biomass increased approximately 3.6 times by supplementation of substrate (OD_600nm_: 0.64 ± 0.03 versus 0.18 ± 0.01). Further elevated pressure impaired the growth of WP3, and the biomass increased merely 2.5 times at 40 MPa (OD_600nm_: 0.42 ± 0.02 versus 0.17 ± 0.01).

Although the growth of WP3 was not significantly affected when pressure increased from 0.1 to 20 MPa, the time course analysis of nitrate concentration suggested that the process of nitrate consumption was influenced by changing of pressure. As demonstrated in Figure 1B, under both pressure conditions, the nitrate concentration in the culture medium decreased slowly at the beginning, followed by a sharp decline that lasted for approximately 4 h during the exponential phase before the nitrate was fully exhausted. Noticeably, the rapid decrease of nitrate took place 8 h after inoculation at 0.1 MPa but 6 h at 20 MPa, suggesting advanced nitrate consumption under elevated pressure conditions.

The *napA* double mutant was cultivated under identical conditions to the wild-type strain. As anticipated, the supplemented nitrate was not utilized, and the growth was not affected (Supplementary Figure S1), which further confirmed that NAP-α and NAP-β were the only systems responsible for nitrate reduction in WP3 under HHP conditions.

### NAP-α or NAP-β Alone Is Sufficient to Confer Nitrate Utilization Under HHP Conditions

Both NAP systems are capable of conducting nitrate respiration under atmospheric pressure conditions (Chen et al., 2011). To illustrate their function under high pressure conditions, growth assays of WP3-α (Δ*napA2*, possesses NAP-α only) and WP3-β (Δ*napA1*, possesses NAP-β only) were performed. As expected, deletion of single *napA* impaired neither cell growth nor maximal biomass at 0.1 MPa (Figures 1C,E). Nevertheless, differences in cellular growth and nitrate consumption were observed between the two mutants and the wild type strain when cultivated at 20 MPa. The growth of WP3-α was clearly improved at elevated pressure (Figure 1D), whereas WP3-β and the wild type strain remained unaffected (Figures 1B,F). In addition, for both WP3-β and the wild type strain, the rapid consumption of nitrate started at 6 h at 20 MPa but 8 h at 0.1 MPa (Figures 1B,F). In contrast, the 2 h differences in nitrate utilization under 0.1 and 20 MPa was not observed in WP3-α. At both pressure conditions, the concentration of nitrate began to decrease at 6 h after inoculation (Figure 1D).

### NAP-β Alone Is Induced by HHP and Nitrate in the WP3 Wild Type

**FIGURE 2:**
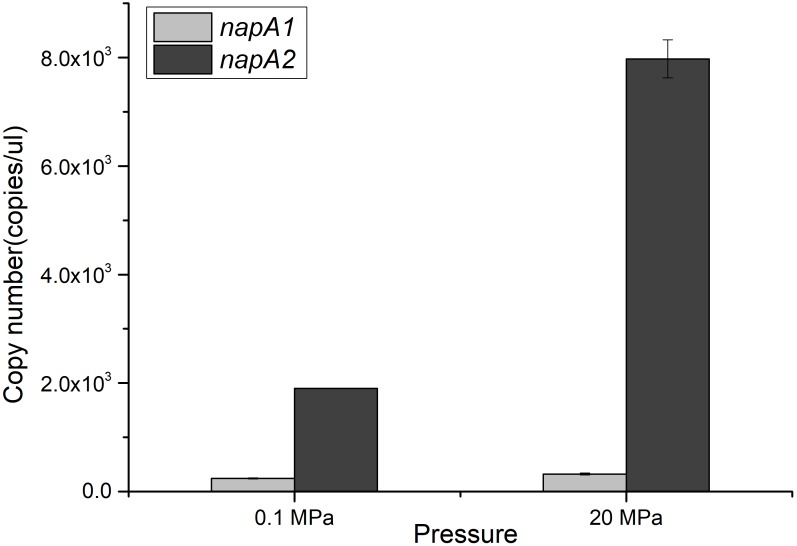
Expression levels of *napA1* and *napA2* in WP3 under different pressure conditions. The copy number of *napA1* and *napA2* in WP3 cells cultivated under different conditions was determined by absolute quantitative RT-PCR. Each value is the average of three measurements.

The divergent profiles of growth and nitrate utilization of the two mutants at 20 MPa led to the presumption that the two NAP systems may have distinct properties and serve different functions during nitrate reduction. To test this hypothesis, we quantified the expression of the two *napA* genes in cDNA samples of WP3 cultivated under different pressure conditions by means of absolute quantitative RT-PCR. As shown in Figure 2, the transcript abundance of *napA2* was approximately one order of magnitude higher than that of *napA1* at both 0.1 MPa (7.81 folds) and 20 MPa (24.7 folds), suggesting that both NAP systems were actively transcribed and that NAP-β took up a higher proportion of nitrate reductase and possibly contributed more to nitrate reduction. In addition, it is noted that the transcript abundance of *napA2* was three times higher at 20 MPa than at 0.1 MPa (7.97 × 10^3^ at 20 MPa versus 1.90 × 10^3^ at 0.1 MPa), while elevated pressure had little effect on *napA1* (3.23 × 10^2^ at 20 MPa versus 2.43 × 10^2^ at 0.1 MPa) (Figure 2).

**Table 2:** Transcriptional analyses of *napA* in WP3 and mutants under different conditions.

Strain	Gene	20E/0.1E	20N/0.1N	0.1N/0.1E	20N/20E	20N/0.1E
WP3	*napA1*	2.03 ± 0.20	1.60 ± 0.42	1.70 ± 0.20	1.34 ± 0.35	2.33 ± 0.31
	*napA2*	3.14 ± 0.50	6.06 ± 1.55	3.65 ± 0.42	7.06 ± 1.81	19.04 ± 2.47
WP3-α	*napA1*	0.61 ± 0.02	2.84 ± 0.10	5.39 ± 0.40	25.10 ± 0.87	15.32 ± 0.53
WP3-β	*napA2*	1.05 ± 0.04	3.40 ± 0.49	7.71 ± 1.16	24.96 ± 3.62	26.18 ± 3.79

*0.1E and 20E represent cells that were cultured in 2216E media under 0.1 or 20 MPa; 0.1N and 20N represent cells that were cultured in 2216E media supplemented with nitrate plus lactate under 0.1 or 20 MPa.*

To better understand the regulation of the two NAP systems in WP3, the effect of substrate and pressure on the transcription of *napA1* and *napA2* were analyzed. As shown in Table 2, supplementation of substrate at both pressure conditions had little effect to the expression of *napA1* (0.1N/0.1E, RQ: 1.70 ± 0.20 and 20N/20E, RQ: 1.34 ± 0.35). Elevated pressure induced the expression of *napA1* slightly only when substrate was absent (20E/0.1E, RQ: 2.03 ± 0.20 and 20N/0.1N, RQ: 1.60 ± 0.42), and its influence was comparable to the collective effect of substrate and elevated pressure (20N/0.1E, RQ: 2.33 ± 0.31). In contrast, both HHP and substrate obviously increase the gene transcription of *napA2* (20E/0.1E, RQ: 3.14 ± 0.50 and 0.1N/0.1E, RQ: 3.65 ± 0.42). The presence of substrate and high pressure condition could further enhance their influence (20N/0.1N, RQ: 6.06 ± 1.55 and 20N/20E, RQ: 7.06 ± 1.81), suggesting a synergistic effect between the two factors. Notably, simultaneous addition of substrate and application of HHP significantly enhanced gene transcription by approximately 19-fold (20N/0.1E). Together, these data suggest that the two NAP systems respond differently to environmental factors at the gene transcription level; the dominant NAP-β was inducible by both substrate and elevated pressure, while NAP-α was constitutively expressed at a relatively lower level.

### The Expression of Two NAP Systems in WP3 Are Correlated

Despite the distinct expression profile of *napA1* and *napA2* in the wild type strain, previous growth assays demonstrated that each NAP system alone was capable of nitrate reduction when the other was mutated. To interpret the discrepancy, we further analyzed the gene expression pattern of *napA1* and *napA2* in single deletion mutants. As shown in Table 2, *napA1* from WP3-α and *napA2* from WP3-β shared similar transcription profiles. HHP induced their expression only when substrate was present (20N/0.1N versus 20E/0.1E), and the addition of substrate obviously augmented gene expression, especially under high pressure conditions (20N/20E and 0.1N/0.1E), suggesting a synergistic effect between elevated pressure and the supplementation of substrate.

Compared to wild type strain, the influence of substrate at both pressure conditions (0.1N/0.1E and 20N/20E) and the collective effect of substrate and HHP (20N/0.1E) to the gene transcription of *napA1* and *napA2* were enhanced in mutant strains. However, the induction by elevated pressure without supplementation of substrate (20E/0.1E) was only observed in the wild-type strain (2.03 ± 0.20 for *napA1* and 3.14 ± 0.50 for *napA2*), but not in the two mutants (0.61 ± 0.02 for *napA1* in WP3-α and 1.05 ± 0.04 for *napA2* in WP3-β). It suggested that the effect of pressure was weakened in single *napA* mutant strains. Moreover, deletion of the other NAP system had a greater effect on the gene expression of *napA1* over *napA2*. Collectively, the results indicated that the expression of the two NAP systems was coupled through an unknown mechanism, and the deletion of one set effected on the remaining set obviously.

### NAP-α Is More Tolerant to Elevated Pressure Than NAP-β

**FIGURE 3:**
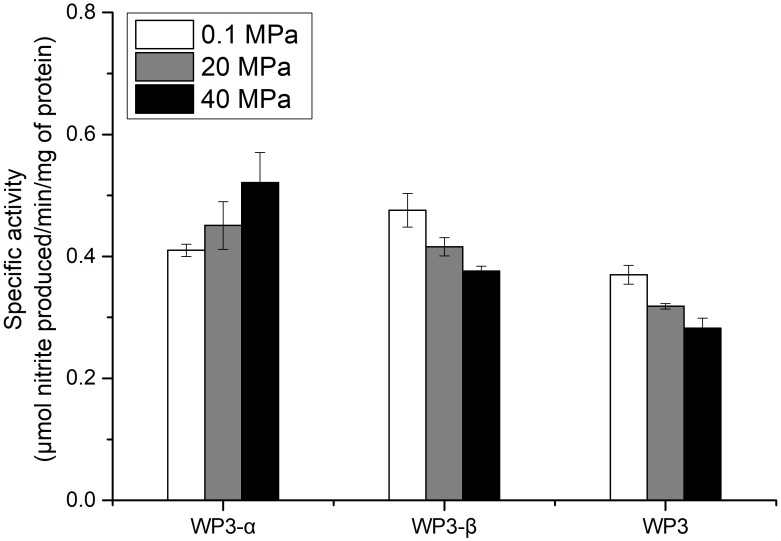
Effect of pressure on the nitrate reductase activity in the periplasmic fractions of WP3 and two *napA* mutants. WP3 and the mutants were cultured at different pressures with the supplementation of substrate. Cells from the mid-exponential phase were harvested, and the periplasmic fractions were obtained. The specific activity of nitrate reductase was measured. Each value is the average of three measurements.

The depth dependent distribution of NAP-α and NAP-β indicated distinct piezotolerance. To analyze the pressure tolerance of individual NAP systems, periplasmic fractions of wild type, WP3-α and WP3-β were prepared from cells cultivated with the presence of nitrate at 0.1 MPa, and the nitrite produced under 0.1, 20, and 40 MPa was measured to estimate the activity of nitrate reductase. As shown in Figure 3, the activity of WP3-α was augmented as the pressure increased, even at 40 MPa. In contrast, the activities of periplasmic fractions from WP3-β and WP3 were reduced at 20 and 40 MPa. Thus, NAP-α was more tolerant to elevated pressure than NAP-β, which is consistent with previous reports that deep-sea species encode NAP-α while shallow water species possess the NAP-β system.

## Discussion

Possessing redundant respiratory terminal enzymes that catalyze identical reduction or oxidation reactions but differ in enzymatic properties is a widely utilized strategy of HHP adaptation in deep-sea bacteria (Tamegai et al., 2005, 2011; Yin et al., 2018). For example, *S. violacea* DSS12 prefers quinol oxidase over cytochrome c4 oxidase under HHP conditions, as the former exhibits higher piezotolerance (Ohke et al., 2013). *Photobacterium profumdum* SS9, *P. phospherum* ANT-2200, and *Vibrio fluvialis* QY27 encode multiple copies of TMAO reductases that are expressed differently in response to elevated pressure (Zhang et al., 2016; Yin et al., 2018). As one of the most extensively studied deep-sea strains, such phenomena have been observed in WP3 as well. Xiong et al. (2016) identified two DMSO respiratory systems: the type I system functions mainly at *in situ* conditions (4°C/20 MPa), and the type II system plays a dominant role under high pressure or low temperature conditions. In this study, we demonstrated that two NAP systems have distinct responses to HHP at both the gene transcription level and enzyme activity level, suggesting different functions and contributions in nitrate reduction in the deep-sea environment.

Although both NAP systems are involved in anaerobic nitrate respiration in WP3, the contribution of each system was unclear. Here, we demonstrated that the absolute expression level of *napA2* was one order of magnitude higher than that of *napA1* (Figure 2). In addition, the periplasmic fraction from the wild type strain exhibited similar piezotolerance as WP3-β, which contained NAP-β only, but obviously different from WP3-α (Figure 3). Taken together, these results suggested that NAP-β is the dominant nitrate reductase in WP3 and possibly played a major role in nitrate reduction.

In this study, we observed that the expression of *napA1* is hardly effected by elevated pressure and nitrate in the wild type strain, while it was clearly induced by both factors when *napA2* was absent (Table 2). It suggested that the expression profile of NAP-α was largely determined by the presence of functional NAP-β. Screening the promoter region of *nap* operons in WP3 revealed binding sites of NarP and EtrA in the upstream region of both *nap* operons and two additional binding sites of Fur (ferric uptake regulator) and Crp upstream of the *nap*-β operon (Supplementary Figure S2). Therefore, we propose that in the wild type strain, the production of functional NAP-β was further promoted by the two additional regulators, and NAP-β alone was sufficient to convert nitrate to nitrite. Consequently, the expression of NAP-α was kept at a relatively lower level, insensitive to the addition of substrate or application of high pressure. On the contrary, inactivated NAP-β due to the deletion of *napA2* led to excessive nitrate, which activated or derepressed the expression of NAP-α.

It should be noted that all the growth experiments were performed at 20°C, the optimal growth temperature of strain WP3. As previously reported, temperature is an important factor that influences the expression of NAP systems. The expression of NAP-α is up-regulated by lower temperature, whereas the expression of NAP-β is reduced at 4°C compared to 20°C (Chen et al., 2011). Therefore, whether NAP-α is constitutively expressed or is substrate and high pressure inducible, as well as its actual proportion in nitrate reductase in the *in situ* environment (4°C, 20 MPa), remain unclear. Further analysis of gene expression and enzyme activity of NAP systems at *in situ* pressure and temperature conditions will be necessary to determine their regulation pattern and contribution to nitrate reduction in the deep-sea environment.

Pressure-induced gene expression was discovered over 20 years ago, yet its mechanism remains unclear for most cases. The NtrBC system in *S. violacea* DSS12 and the ToxRS system in *P. profumdum* SS9 are two of the most extensively studied pressure-related regulators (Welch and Bartlett, 1998; Bidle and Bartlett, 2001; Nakasone et al., 2002). In *S. violacea* DSS12, elevated pressure increases the intracellular level of NtrC through an unknown mechanism and promotes the binding of NtrC to the enhancer region, thus up-regulating the expression of the target gene (Nakasone et al., 2002). The ToxRS regulatory system is presumed to sense membrane changes resulting from HHP and regulate the expression of two dozen genes (Campanaro et al., 2012). However, the binding sites of NtrBC and ToxRS are not present in the promoter region of the NAP systems in WP3, suggesting that a yet-to-be-discovered mechanism may be involved in the pressure-induced expression of *nap* operons. Considering that both the NtrBC and ToxRS systems are not specifically found in deep-sea species, it is possible that known regulators of NAP systems could have evolved the ability to sense pressure in WP3 as well. NarQ-P is the typical two-component system that is responsible for the substrate induction of the NAP system (Stewart, 2003). In addition, several global regulators, such as Fur, Crp and EtrA, are also known to be involved in the regulation of *nap* in *Shewanella* (Cruz-Garcia et al., 2011; Dong et al., 2012; Yang et al., 2013). Whether any of these regulators take part in the pressure regulation of *nap* operons in WP3 is worth further investigation.

Examination of the enzymatic activity of single *napA* mutants under different pressure conditions showed that NAP-α is more tolerant to high pressure than NAP-β (Figure 3). These results perfectly explain why the WP3-α strain exhibited better growth and higher final biomass than WP3-β and WP3 at 20 MPa (Figure 1). The better piezotolerance of NAP-α was also consistent with the observation that only the NAP-α system was present in the genomes of strains isolated from a depth of over 5,000 m, such as *S. violacea* DSS12 (5,110 m), *S. benthica* KT99 (9,856 m), and *S. benthica* DB21MT-2 (10,898 m), whereas some shallow water species encode NAP-β only (Supplementary Figure S3).

It is well-known that differences in the amino acid composition and protein structure could result in different tolerances to pressure (Ohmae et al., 2004; Hamajima et al., 2016). The amino acid sequences of NapA1 and NapA2 share 71% identity, and it has been proposed that the two NapA proteins evolved separately (Wang and Chen, 2015). Apart from the differences in their sequences, the components of the two NAP systems differ as well. NAP-α is composed of four subunits, NapD, A, B, and C, while NAP-β is composed of three subunits, NapD, A, and B. Instead of using NapC, which specifically transfers electrons to NapAB, the NAP-β system uses CymA which delivers electrons not only to NapAB for nitrate reduction but also to NrfA for nitrite reduction (Gao et al., 2009; Chen et al., 2011). It is thus believed that NAP-α is more efficient at performing nitrate reduction than NAP-β (Chen et al., 2011). The discrepant composition and physiological function between the two NAP systems could also be a reason for their differences in piezotolerance.

## Conclusion

We confirmed that two sets of NAP systems were involved in the nitrate utilization of WP3 under HHP and that each NAP systems alone was sufficient to confer nitrate-sustained growth under HHP. When cultivated under optimal pressure and temperature conditions (0.1 MPa, 20°C), NAP-β, the substrate and pressure inducible nitrate reductase system, plays the dominant role in nitrate reduction. However, NAP-α exhibited better tolerance to elevated pressure. Collectively, our results suggest that the deep-sea bacteria *S. piezotolerans* WP3 may benefit from duo-NAP systems with distinct properties and functions to cope with the ever-changing physical and chemical parameters in the deep-sea environment where it dwells.

## Author Contributions

X-GL, W-JZ, and L-FW designed the study, analyzed the data, and wrote the manuscript. X-GL, TJ, and H-ZT performed the experiments. XX, H-HJ, and X-QQ provided the technical support. All the authors read and approved the final manuscript.

## Conflict of Interest Statement

The authors declare that the research was conducted in the absence of any commercial or financial relationships that could be construed as a potential conflict of interest.
