# Supplementary material for: Pressure-Regulated Gene Expression and Enzymatic Activity of the Two Periplasmic Nitrate Reductases in the Deep-Sea Bacterium Shewanella piezotolerans WP3
Source: Front Microbiol. 2018 Dec 21;9:3173. doi: 10.3389/fmicb.2018.03173 (PMC6308320; doi:10.3389/fmicb.2018.03173)
Supplement: Supplementary file 1 [file Table_1.DOCX]

Supplementary Material

Pressure-regulated gene expression and nitrate reductase activity of periplasmic nitrate reductases in the deep-sea bacterium *Shewanella piezotolerans* WP3

**Xue-Gong Li^1,2^, Wei-Jia Zhang^1,2*^, Xiang Xiao^3,4^, Hua-Hua Jian^3^, Ting Jiang^1,5^, Hong-Zhi Tang^1,5^, Xiao-Qing Qi^1,2^, Long-Fei WU^2,6^**

*** Correspondence:** Wei-Jia Zhang: [wzhang@idsse.ac.cn](mailto:wzhang@idsse.ac.cn)

## Supplementary Figures


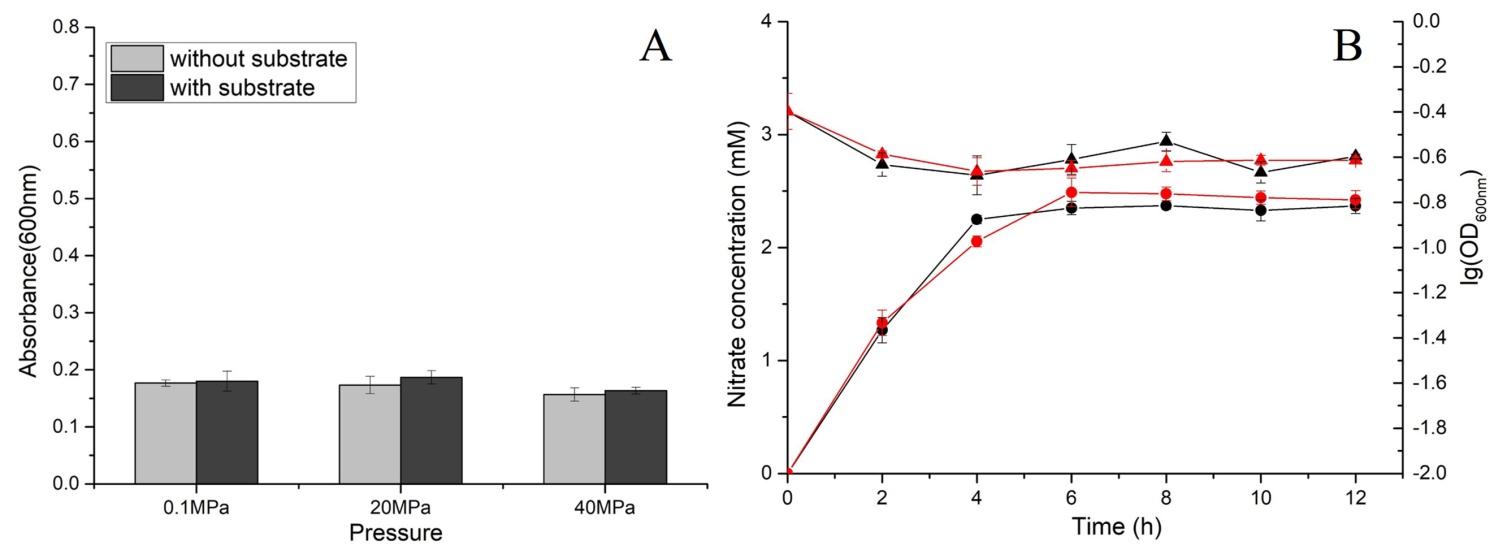


**Figure S1 |** Cell growth and nitrate consumption curve of duo-*napA* deletion mutant under different pressures.

Final biomass of duo-*napA* deletion mutant cultivated in 2216E media with or without substrate (nitrate plus lactate) at different pressure conditions (A). Growth curve and nitrate consumption curve of duo-*napA* deletion mutant at 0.1 MPa and 20 MPa (B). Each value was the average of three measurements. ●: cell density at 0.1 MPa; ●: cell density at 20 MPa; ▲: nitrate concentration of the supernatant at 0.1 MPa; ▲: nitrate concentration of the supernatant at 20 MPa.


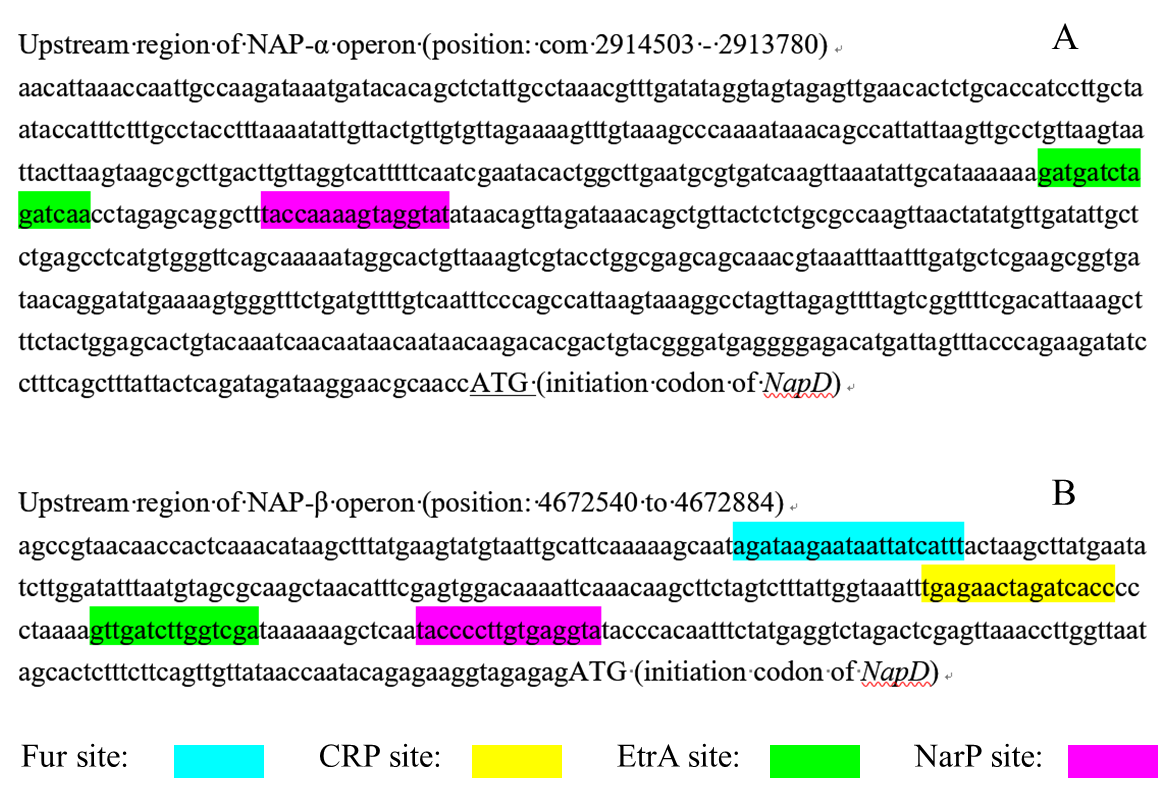


**Figure S2 |** Putative transcriptional regulator analyses of NAP systems in WP3.

Upstream regions of NAP-α (A) and NAP-β (B) in WP3.


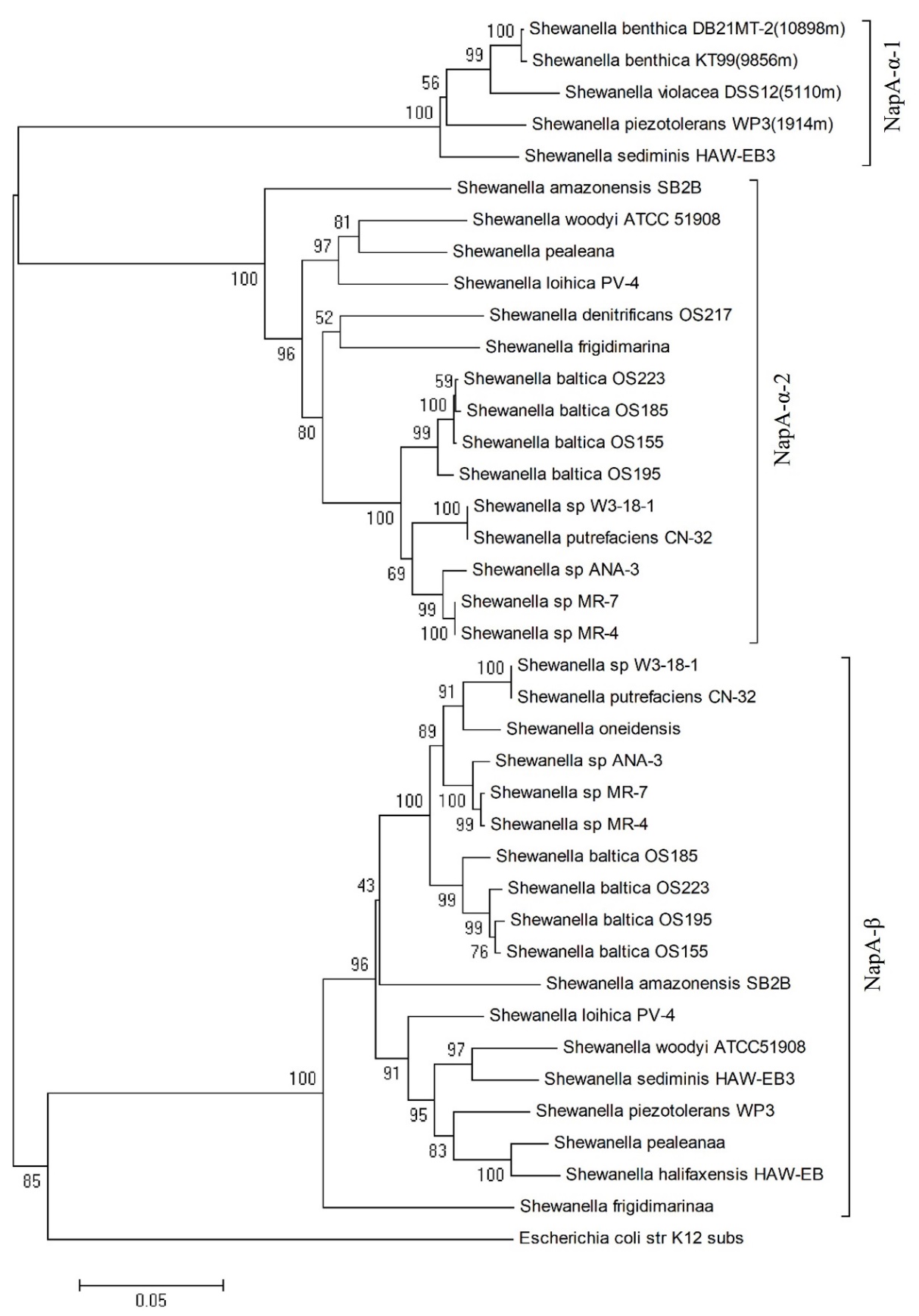


**Figure S3 |** Phylogenetic tree constructed based on NapA protein sequences.

Tree topography and evolutionary distances were determined using the neighbor-joining method with 1,000 replicates of bootstrapping. NapA of *E. coli* was used as the out-group.
